# Development and Evaluation of an Integrated Nutritional Health Care Information System

**DOI:** 10.1055/a-2723-6679

**Published:** 2025-11-20

**Authors:** Yuner Chen, Mu Li, Weijie Xie, Xiuxia Feng, Huishu Tian, Wenhui Wang

**Affiliations:** 1 Information and Data Centre, Guangzhou First People's Hospital, Guangzhou Medical University, Guangzhou, China; 2 Information and Data Centre, the Second Affiliated Hospital, School of Medicine, South China University of Technology, Guangzhou, China; 3 Department of Information, Nanfang Hospital, Southern Medical University, Guangzhou, China

**Keywords:** nutritional health care information system, revised DeLone and McLean IS success model, reliability and validity, nutritional diagnosis and treatment, cross-professional and cross-sectoral model

## Abstract

**Background:**

Malnutrition remains a critical global health challenge among hospitalized patients, necessitating effective nutrition support systems.

**Objectives:**

This study aims to construct and evaluate a cross-professional, cross-sectoral nutrition health care information system (CPCS-NHIS) to optimize clinical nutrition management.

**Methods:**

The system integrates modified medical systems to unify information flows, knowledge, and clinical roles. In addition, a 20-item across six dimensions electronic questionnaire based on the revised DeLone and McLean IS Success model was developed to evaluate the success of the CSCP-NHIS. Besides, user satisfaction was assessed as a key dimension using a 5-point Likert's scale (1 = strongly disagree to 5 = strongly agree). Finally, the results of the questionnaire were further validated by reliability and validity analysis.

**Results:**

The CPCS-NHIS features comprehensive functions including bedside nutritional screening, assessment, intervention, diagnosis, monitoring, enteral nutrition prescription, and QR-code autopayment. Over 80% of users expressed willingness to continue using and recommending the system. The questionnaire results demonstrated strong psychometric properties: all Cronbach's α coefficients >0.80, confirmed reliability; confirmatory factor analysis showed convergent validity (the average variance extracted >0.50, construct reliability >0.80); and model fit indices were excellent, with a chi-square value of 1.86, a root mean squared error of approximation of 0.09, a root mean square residual of 0.02, and a comparative fit index of 0.9.

**Conclusion:**

The CPCS-NHIS provides a practical framework for existing nutritional health care information systems, based on the nutrition care process and model, with robust psychometric evidence and high user acceptance.

## Background and Significance

Malnutrition, defined as any form of nutritional imbalance, 1 poses a significant health challenge in hospitalized adults worldwide, with prevalence rates ranging from 20% to 50% 2 3 It is particularly prevalent in patients in the elderly, 4 in those with cancer, 5 gastrointestinal diseases (e.g., inflammatory bowel disease, short bowel syndrome), liver cirrhosis, chronic kidney disease 6 and various forms of pancreatitis, 7 and has been associated with some adverse effects, such as poor surgical outcomes, reduced quality of life, increased mortality, high hospital costs, prolonged hospitalization, and frequent readmission. 2 8 9 10 11 With societal advancements, the role of nutrition support therapy in clinical practice is increasingly recognized as a critical component of patient care, 12 aimed at improving the nutritional status and health of patients. This underscores the urgent need for the development of an effective nutritional health care information system (NHIS) in early and appropriate nutritional interventions and management.

The Nutrition Care Process (NCP), a standardized framework comprising assessment, diagnosis, intervention, and monitoring/evaluation, guides dietitians in delivering evidence-based care. 13 However, many small hospital institutions, including small community hospitals, medical clinics in developing nations, and specialized hospitals, face systemic barriers to operationalizing the NCP due to limited integration of health information technology. Prior to digitization, all nutritional prescriptions, assessment/screening records, etc. were recorded using paper records, hindering efficient information exchange, leading to inconsistent dietary recommendations and treatments. In addition, conventional NHIS often operate in a fragmented manner that limiting data sharing 14 and collaboration 15 16 17 among dietitians, clinicians, nurses, and administrators. Outdated practices—such as manual inventory trackings 18 or paper-based prescriptions—hinder efficient information exchange, leading to inconsistent dietary recommendations and treatment. These challenges underscore the need for a unified NHIS to support seamless NCP implementation across disciplines. 19

While progress has been made in hospital NHIS, existing systems have limitations. For example, Mirtallo et al. 20 proposed a web-based application for managing parenteral nutrition patients and medical prescriptions. Chen et al. 21 utilized expert system techniques in artificial intelligence to assist chronic kidney disease patients undergoing hemodialysis, resulting in a web-based expert system for nutritional diagnosis by dietitians. Taweel et al. 22 designed a distributed system for home care services, offering features like activity monitoring and nutritional reasoning for older individuals. Kuo et al. 23 developed a nutritional information system for cancer patients, aiding dietitians in data entry and analysis for tailored nutritional interventions. These solutions, although innovative, are often disease- or department-specific, lacking the interoperability needed for standardized, hospital-wide NCP workflows. This results in fragmented data ecosystems, incompatible workflows, and missed opportunities for holistic patient care. 16

However, cross-professional, cross-sectoral collaboration has shown promise in addressing such gaps. For instance, multidisciplinary teams involving dietitians, nurses, and physicians have improved nutritional outcomes in postoperative patients by 30% through coordinated care, 24 whereas volunteer-assisted feeding programs have doubled patient intake and reduced nursing time. 25 These collaborations involve systems of data sharing and integrated workflow systems, but their success has not been evaluated and verified, posing problems for user satisfaction, compatibility, and consistency across hospitals.

Together, prior works highlight the demand for reliable, integrated, and evidence-based clinical informatics solutions, to enhance standardized nutrition care and mitigate hospital malnutrition.

## Objectives

This study aims to design, develop, and evaluate a cross-professional, cross-sectoral nutrition health care information system (CPCS-NHIS) to address data fragmentation and workflow challenges in hospital nutrition management. Unlike conventional NHIS, the CPCS-NHIS integrates multiple medical systems to support the entire nutrition management process—encompassing risk screening, assessment, intervention, program-assisted generation (which enables dietitians to create intervention plans by selecting predefined items (e.g., tests, dietary protocols) from a knowledge base, streamlining the NCP), diagnosis, prescriptions, electronic nutrition medical records, and code payment all related to nutrition—while preserving clinical users' existing workflows. By fostering collaboration across dietitians, clinicians, nurses, dispensers, and administrators, the system seeks to standardize and enhance nutrition care delivery. The revised DeLone and McLean IS Success Model 26 is employed to evaluate the CPCS-NHIS's success across six dimensions such as system quality, information quality, service quality, user satisfaction, intention to use and net benefits, assessing its potential to optimize clinical nutrition management.

## Methods

### System Design

**Fig. 1:**
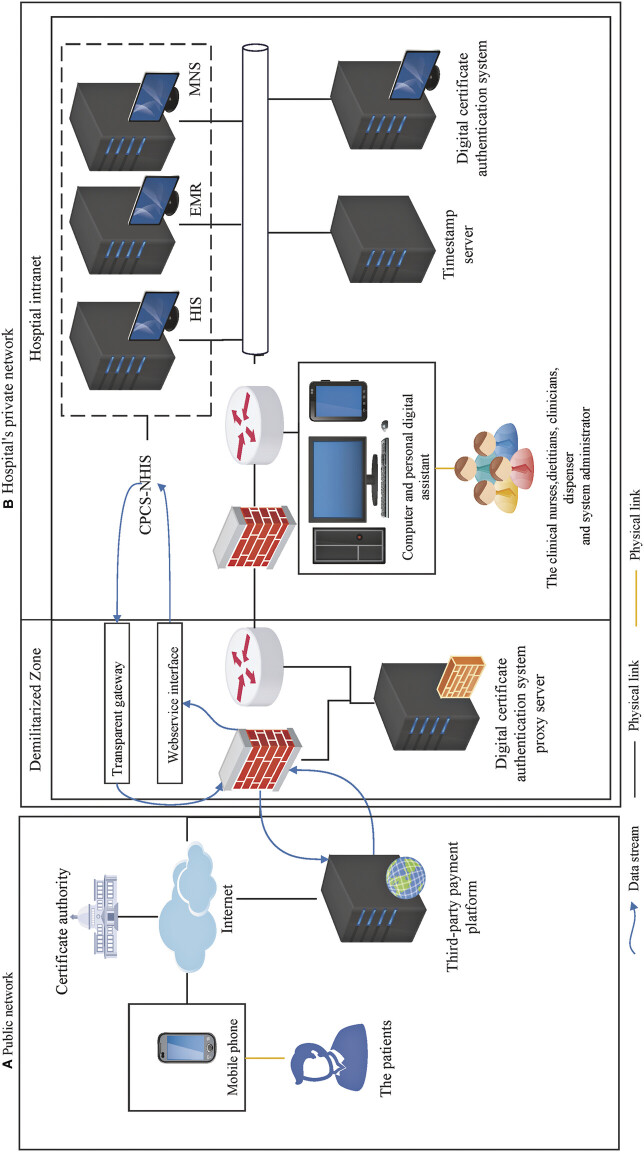
The deployment diagram of the CPCS-NHIS. ( **A** ) Public network. ( **B** ) Hospital private network. CPCS-NHIS, cross-sectoral nutrition health care information system.

The study was conducted at a comprehensive tertiary hospital, which has established a national reputation in gastrointestinal and anal specialties, with a dedicated Nutrition Department and multidisciplinary staff, including dietitians, nurses, clinicians, and IT specialists, who collaborated to develop the CPCS-NHIS. The CPCS-NHIS was designed based on the NCP, 13 a standardized framework comprising nutrition assessment, diagnosis, intervention, and monitoring/evaluation. This process requires seamless collaboration across disciplines and rapid data integration. Based on insights gained from more than 20 discussions(1–2 hours each time)—including brainstorming sessions, 27 focus groups, 28 and seminars—with dietitians, clinicians (gastrointestinal, colorectal, oncology), nurses, department heads (nursing, medical, information), IT specialists, and staff in the finance department, we developed the CPCS-NHIS to align with local hospital workflows through on-premises deployment on hospital servers, collaboration with clinical nutrition departments to ensure compatibility with existing practices, and ongoing maintenance by the hospital's IT team to support operational continuity. Fig. 1 illustrates the CPCS-NHIS deployment, showing data streams (blue arrows), physical links (orange straight lines) and network links (black straight lines) between patients, staff, and system modules. For the hospital's data integration and storage, the methods such as webservices and transparent gateways enable data exchange across clinical systems, storing the data in the database, and a disaster recovery strategy with off-site backups ensures data security and availability.

To minimize the learning cost (such as learning time, energy, etc.) for medical staff on the new system and improve patient care processes, CPCS-NHIS integrates with existing hospital systems—hospital information system (HIS), electronic health record (EHR) system, and mobile nursing system (MNS)—while introducing 14 key modifications ( Table 1 ) to support NCP-aligned functions: bedside nutritional risk screening, assessment, intervention, program-assisted generation (creating NCP intervention plans by offering predefined options from a knowledge base of tests and protocols), prescription, electronic nutrition medical records, program reminders (for timely and automatic recommendation of nutritional interventions), and automated payment coding (system's generation of a QR-coded prescription/payment form for enteral nutrition orders). These modifications were selected through a needs assessment involving gap analyses of existing hospital systems against clinical nutrition workflow requirements, informed by user interviews with dietitians, nurses, and clinicians. Each modification addresses either missing functionalities (e.g., NCP assessment tools) or interoperability barriers (e.g., data exchange between HIS and EHR systems), prioritizing solutions that resolve frequent clinician-reported issues, minimize workflow disruptions, and enhance care coordination. Moreover, these functions interconnect through the hospital's network architecture, which utilizes a demilitarized zone to ensure secure data exchange between public and private networks. Clinicians and staff access the system through intranet login with digital certificate authentication, whereas patients use internet-based portals for convenience. Notably, since the medical staff were familiar with the original systems, this may enable them to effectively collaborate across professions and sectors.

**Table 1:** Priority modifications of the hospital information system, electronic health record system, mobile nursing system, and third-party payment platforms

Modified system	Modified content
HIS a	1. Add a “Nutritional Prescribing” tab with access rights control, allowing only authorized users to access it. Include nutritional prescription pads/orders with QR codes for billing, as required by hospital management 2. Establish a new virtual pharmacy for nutritional preparations 3. Automatically retrieve nutritional intervention plans from the EHR system and add “screened” and “malnourished” markers to the patient list to indicate their status 4. Upon formulating nutritional intervention plans, first-time users click the “Open” button on the nutritional prescribing interface to display the plans 5. Add a “Nutritional Plan” button to the nutritional prescription interface to enable clinicians to proactively view nutritional intervention plans 6. Synchronize nutrition order information with a third-party payment platform via webservice interface to facilitate transactions
EHR b system	1. Added Tools and Forms: Nutritional Risk Screening Tool (NRS2002), 29 Overall Subjective Scale, 30 PG-SGA, 31 c New human nutrition rating method-MNA, 32 d and Nutrition Intervention Programme Evaluation Form. Structured storage and automatic score calculation functions are included 2. Maintain a knowledge base related to nutritional risk screening and testing. Include information regarding nutritional drugs and food packages, intervention modalities, and enteral/parenteral medical orders
MNS e	1. Automatically access EHR-maintained nutritional risk screening form templates and completed form contents 2. Add auto-calculation functionality for points on forms 3. Synchronize nutritional risk screening forms with the EHR system
Third-party payment platform	1. After patients scan the payment QR code generated by the third-party payment platform for prescription pads/orders and complete the payment, the payment platform accesses the Webservice interface of the HIS and submits the payment information 2. Support and process patient refunds and synchronize the status of charged/refunded/shipped orders with the HIS 3. Provide a transparent gateway for the HIS to view detailed information regarding charges for nutritional drugs/foods

a HIS represents hospital information system.

b EHR represents electronic health record.

c PG-SGA represents the patient-generated subjective global assessment.

d MNA represents the mini-nutritional assessment.

e MNS represents mobile nursing system.

### Performance Evaluation

The performance of CPCS-NHIS was evaluated using the revised DeLone and McLean IS Success Model, 26 33 which assesses six interrelated dimensions: System Quality (e.g., ease of use, integration), Information Quality (e.g., accuracy, timeliness), Service Quality (e.g., responsiveness), System Use (e.g., frequency), User Satisfaction (e.g., perceived usefulness), and Net Benefits (e.g., workflow efficiency). Among them, System, Information and Service Quality drive Use and Satisfaction, which mediate Net Benefits. These six dimensions were further constructed as a 20-item questionnaire, with items derived from literature 34 35 36 37 and tailored to CPCS-NHIS. For example, System quality, which includes factors such as ease of use, flexibility, customizability, maintainability, and integration, is the key to the efficiency of system operation. Therefore, we designed the corresponding questionnaire questions based on this element: the system's functional interface is friendly and easy to understand. Each item was rated on a 5-point Likert scale 38 (1 = strongly disagree, 5 = strongly agree). Table 2 details the evaluation indices and items.

**Table 2:** Evaluation indices and evaluation items of the cross-sectoral nutrition health care information system

Evaluation dimension	Evaluation index	Thematic number	Evaluation item
System quality	Ease of use	N1	The system's functional interface is user-friendly and easy to understand
	Flexibility	N2	Flexible configuration grants departments authorization to prescribe enteral nutrition through the system interface
	Customizability	N3	The system supports customization and personalization of the nutritional risk screening form and the content of the statistical nutritional screening score form
	Maintainability	N4	The system supports the maintenance of nutritional plans, nutritional drugs, nutritional food instructions, and other related content
	Integration	N5	The system integrates information from multiple systems (e.g., HIS, EHR system, MNS) for clinical access
Information quality	Accuracy	N6	The system provides accurate labelling feedback; for example, if the total nutritional risk screening score is ≥3, the patient is labelled as “screened”
	Timeliness	N7	Clinical nurses, dietitians, and clinicians are satisfied with the comprehensiveness and timeliness of the information provided by the system regarding tests, examinations, and other elements of the nutrition intervention plan
	Relevance	N8	The system has clear identity labels for patients who have undergone nutritional risk screening and assessment; these labels can be used to determine the current nutritional statuses of patients
	Perceived usefulness	N9	The prompts provided by the system are helpful, and the information recorded by the system assists in clinical research or teaching
Service quality	Reliability	N10	The system operates stably
	Responsiveness	N11	Clinicians can promptly prescribe enteral nutrition through the system, thereby saving time
	Credibility	N12	The system records and traces user operations
	System maintenance	N13	The system can be continuously optimized and improved in accordance with user needs
User satisfaction	Overall satisfaction	N14	Overall, you are satisfied with the system
	Satisfaction with decision support	N15	The scoring form created by the system and the calculation of the screening risk score are useful for evaluating and making decisions regarding patients' nutritional status
Intention to use	Empathy	N16	You are willing to continue using the system
		N17	You are willing to recommend the system to others
Net benefits	Individual effect	N18	The system assists clinical nurses, dietitians, and clinicians in making effective decisions regarding patients' treatment
		N19	The system improves the clinical efficiency of nurses, dietitians, and clinicians
	Organizational effect	N20	The system can enhance medical benefits

Abbreviation: EHR, electronic health record.

### Statistical Analysis

Data analysis was conducted using the Scientific Platform Serving for Statistics Professional (Shanghai Zhongyan Technology Co., Ltd., Shanghai, China). To assess questionnaire reliability, Cronbach's α was calculated for internal consistency across the six dimensions. The Kaiser–Meyer–Olkin (KMO) measure and Bartlett's test were used to confirm suitability for factor analysis. Confirmatory factor analysis evaluated convergent validity and structural model fit. Descriptive statistics, including means and standard deviations, were computed to summarize responses.

## Results

### System Functionality of Cross-Sectoral Nutrition Health Care Information System

**Fig. 2:**
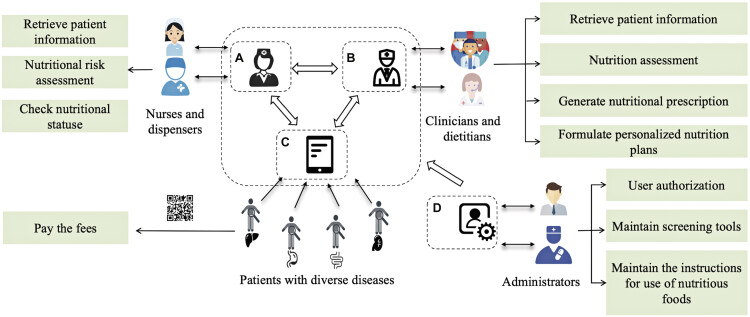
Core functionalities of CPCS-NHIS modules. ( **A** ) The nurse workstation. ( **B** ) The doctor workstation. ( **C** ) The patient terminal. ( **D** ) The system administration. CPCS-NHIS, cross-sectoral nutrition health care information system.

The main results for the functional construction of the CPCS-NHIS are presented in Fig. 2 and Appendix 1 (available in the online version only). As shown in Fig. 2 , the CPCS-NHIS consists of four modules: nurse workstation, doctor workstation, patient terminal, and system administration and is designed to support the steps of the NCP: assessment, diagnosis, intervention, and monitoring/evaluation.

The nurse workstation ( Fig. 2A ) enables authorized nurses and dispensers to access patient data on demand, conduct nutritional risk screenings and track patient statuses in real-time, supporting NCP assessment. The doctor workstation ( Fig. 2B ) allows clinicians and dietitians to access patient data, perform nutrition assessment, retrieve test results, and generate tailored nutrition plans and prescriptions, facilitating NCP assessment, diagnosis, intervention, and monitoring. The patient terminal ( Fig. 2C ) provides internet-based access for patients to process payments via QR code scanning, enhancing engagement in their care. The system administration module ( Fig. 2D ) manages access control, integrates screening tools, ensures secure authentication, and maintains nutritional guidelines, supporting all NCP steps.

Integration with HIS, EHR, MNS, and third-party payment platforms required minimal modifications, with transformation workloads of 1, 0.5, 0.5, and 0.7 person-months, respectively ( Table 1 ). This approach preserved clinical workflows while enabling cross-professional collaboration among nurses, dietitians, clinicians, dispensers, and administrators.

### Subdivided Functions in Nutritional Care

**Fig. 3:**
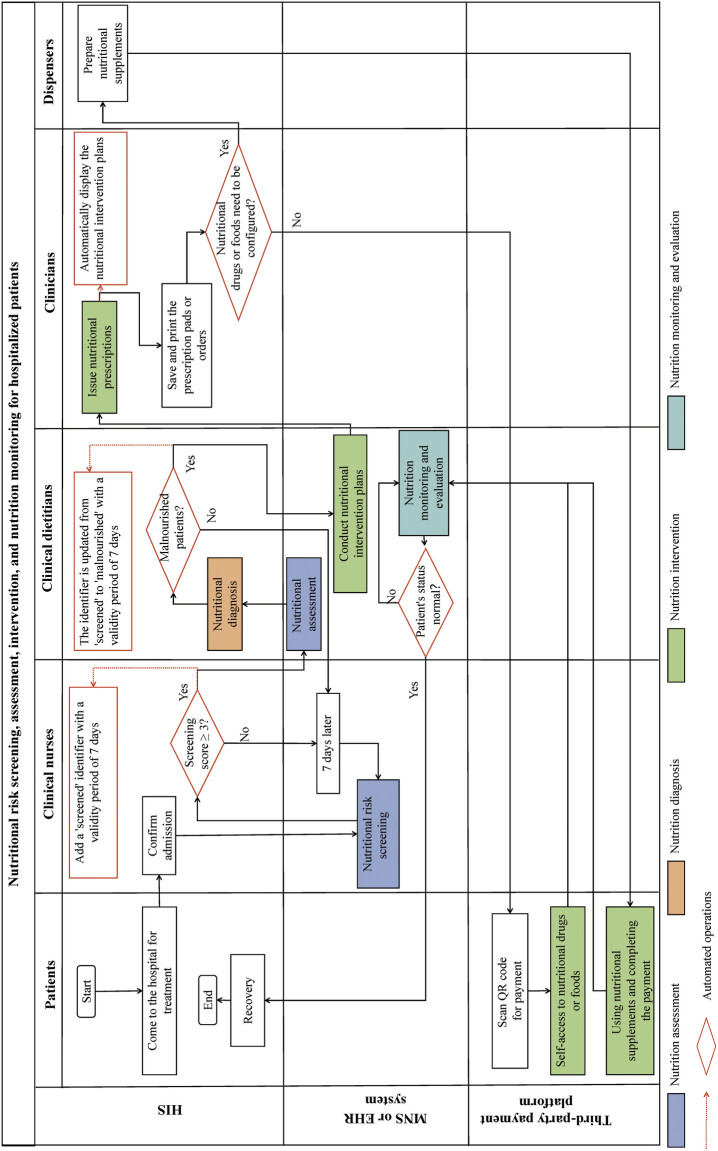
CPCS-NHIS workflow, aligned with the Nutrition Care Process steps. Blue block: assessment, orange block: diagnosis, green block: intervention, cyan block: monitoring/evaluation. The red dotted arrows and borders indicate automated operations within the HIS that require no human intervention. CPCS-NHIS, cross-sectoral nutrition health care information system; HIS, hospital information system.

Fig. 3 presents the inpatient nutrition care workflow in the CPCS-NHIS, with color-coded identification for the embedded NCP's four steps (blue for nutrition assessment, orange for nutrition diagnosis, green for nutrition interventions, and cyan for nutrition monitoring and evaluation). Each module supports specific NCP steps, e.g., the nurse module facilitates assessment by nutritional screening, whereas the doctor module aids diagnosis with decision-support tools. Five actors are involved in the CPCS-NHIS: clinical nurses, dietitians, clinicians, patients, and dispensers. Detailed descriptions of workflow node are provided in Fig. 3 , where the red dotted arrows and borders indicate automated operations within the HIS that require no human intervention.

#### Nutritional Risk Screening and Assessment

According to the study hospital's regulations, nurses perform nutritional risk screenings for patients by logging into the MNS on a personal digital assistant or EHR system on a computer within the first 7 days of hospitalization. The system automatically calculates and updates the total screening score based on inputs from the nurse's nutritional risk screening checklist and reevaluates it every 7 days. If the total screening score is ≥3, the system marks these patients with a “screened” identifier in the HIS, enabling clinical dietitians to quickly identify patients who require a nutritional assessment.

#### Nutritional Diagnosis and Development of a Nutritional Intervention Plan

Clinical dietitians conduct nutritional assessments for patients marked as “screened.” By synthesizing multidimensional clinical data—including medical records, physical examination findings, nutritional risk screening results, laboratory analyses, and ancillary assessments—the dietitian systematically identifies nutritional deficits/imbalances and their etiology, formulates a standardized nutrition diagnosis using NCP terminology. If the assessment and diagnosis indicate a need for nutritional intervention, dietitians can swiftly select appropriate nutritional examinations, tests, and medical advice from a premaintained knowledge base. They then formulate an individualized nutritional intervention plan for the patient. After this plan is developed, the patient's status in the HIS is updated from “screened” to “malnourished” to indicate that the patient is experiencing malnutrition and to alert clinicians that the patient needs to receive nutritional intervention accordingly.

#### Nutrition Intervention Plan Prompting and Prescription

When a clinician authorized to allow nutritional prescribing issues a nutritional order for a patient with malnutrition identified as “malnourished” for the first time, the HIS automatically displays the relevant nutritional intervention plan for the clinician's reference. In addition, clinicians can access this plan by clicking the “Nutritional Plan” button on the nutritional prescription interface.

#### Patient Payment

For ready-to-consume nutritional products, such as prepackaged items, patients may directly collect their nutrition medications or food from the Clinical Nutrition Department after scanning the payment code on their prescription pads or orders and completing the transaction. If the clinician prescribes a nutritional preparation, the Configuration Unit prepares and delivers this preparation to the ward for the patient's use. Once the nutrition product is obtained, the patient can scan the QR code on the prescription or order to process the payment.

#### Nutrition Monitoring and Evaluation

After implementing nutritional therapy, the clinical dietitian employs a comprehensive monitoring framework to evaluate whether the patient's nutritional status has improved and whether further therapy is required. This framework integrates clinical parameters (e.g., anthropometric measurements, biochemical indicators), dietary intake patterns (e.g., energy balance, macronutrient/micronutrient profiles), and health status indicators (e.g., comorbidities, treatment adherence).

### Questionnaire Results for Cross-Sectoral Nutrition Health Care Information System Performance

We collected a total of 100 completed and submitted questionnaires (the participants gave informed consent) from users of the CPCS-NHIS, including 38 nurses, 34 clinicians, 14 dietitians, 7 dispensers and 7 administrators, with 43 responses from males (mean age: 34.36 ± 8.40 years) and 57 from females (mean age: 34.16 ± 8.50 years). This distribution reflects the CPCS-NHIS's cross-professional user base, ensuring diverse perspectives on system performance. Table 3 shows the details of mean scores and standard deviations for the different user groups, and the users satisfaction. For instance, in System Quality items, the item on access control (N2) received an overall mean score of 4.22 ± 0.73, with clinicians rating it lowest (4.12 ± 0.63), and user satisfaction was 84% . For Information Quality items, such as accurate patient identification for NCP assessment (N6), averaged 4.32 ± 0.71, with clinical nurses reporting strong ratings (4.29 ± 0.6), user satisfaction was 91%. Service Quality, including traceability of operations (N12), received a mean of 4.3 ± 0.64, with administrators noting consistent performance (4.71 ± 0.45), user satisfaction was 90%. For User Satisfaction items, like nutritional risk screening forms aiding NCP decision-making (N15), averaged 4.36 ± 0.66, with dietitians rating it highest (4.5 ± 0.5), user satisfaction was 92%. For Intention to Use items, with at least 82% of participants willing to continue using and recommend CPCS-NHIS in N16 (4.36 ± 0.69) and N17(4.11 ± 0.92). For the Net Benefits items, such as support for clinical judgments (N18), scored 4.26 ± 0.72, with clinical nurse rating workflow integration highly (4.24 ± 0.63), and user satisfaction was 88% . These questionnaire results ( Table 3 ) showed that the average score for each of the 20 items assessing the CPCS-NHIS exceeded 4.0 on a 5-point Likert scale, indicating high ratings across the six dimensions of the revised DeLone and McLean IS Success Model.

**Table 3:** Distribution of participants' scores across 20 evaluation items

Evaluation dimension	Thematic number	Mean ± SD for user groups					Mean ± SD ( *n* = 100)	Satisfaction rate	Percentage of neutral to strongly agree
		Clinical nurse ( *n* = 3 8)	Clinicians ( *n* = 34)	Dietitians ( *n* = 14)	Dispensers ( *n* = 7)	Administrators ( *n* = 7)			
System quality	N1	4.18 ± 0.76	4 ± 0.77	4.43 ± 0.73	4.14 ± 0.83	4.14 ± 0.64	4.15 ± 0.77	81	98
	N2	4.24 ± 0.74	4.12 ± 0.63	4.43 ± 0.9	4.29 ± 0.7	4.14 ± 0.64	4.22 ± 0.73	84	99
	N3	4.16 ± 0.67	4.24 ± 0.81	4.21 ± 1.08	4.29 ± 0.7	4.57 ± 0.49	4.23 ± 0.79	87	97
	N4	4.18 ± 0.68	4.24 ± 0.69	4.57 ± 0.49	4.29 ± 0.7	4.43 ± 0.49	4.28 ± 0.67	88	100
	N5	4.21 ± 0.66	4.09 ± 0.82	4.21 ± 0.67	4.57 ± 0.73	4.43 ± 0.49	4.21 ± 0.73	84	99
Information quality	N6	4.29 ± 0.6	4.18 ± 0.86	4.79 ± 0.41	4 ± 0.53	4.57 ± 0.49	4.32 ± 0.71	91	99
	N7	4.26 ± 0.68	4.03 ± 0.86	4 ± 0.65	4.14 ± 0.64	4.29 ± 0.45	4.14 ± 0.74	83	98
	N8	4.29 ± 0.68	4.21 ± 0.72	4.5 ± 0.63	4.43 ± 0.73	4.57 ± 0.49	4.32 ± 0.69	89	99
	N9	4.34 ± 0.66	4.21 ± 0.76	4.14 ± 0.83	3.86 ± 0.64	4.57 ± 0.49	4.25 ± 0.73	85	99
Service quality	N10	4.32 ± 0.61	4.24 ± 0.77	3.86 ± 0.91	4.43 ± 0.73	4.29 ± 0.7	4.23 ± 0.75	83	99
	N11	4.18 ± 0.76	4.12 ± 0.76	4.21 ± 0.86	4.43 ± 0.73	4.43 ± 0.49	4.20 ± 0.77	81	99
	N12	4.34 ± 0.66	4.18 ± 0.66	4.36 ± 0.48	4.14 ± 0.64	4.71 ± 0.45	4.30 ± 0.64	90	100
	N13	4.29 ± 0.65	4.18 ± 0.82	4.21 ± 0.56	4.14 ± 0.64	4.57 ± 0.49	4.25 ± 0.70	87	99
User satisfaction	N14	4.21 ± 0.61	4.15 ± 0.77	4.5 ± 0.63	4.29 ± 0.7	4.57 ± 0.49	4.26 ± 0.69	86	100
	N15	4.37 ± 0.67	4.26 ± 0.7	4.5 ± 0.5	4.43 ± 0.73	4.43 ± 0.49	4.36 ± 0.66	92	99
Intention to use	N16	4.42 ± 0.63	4.26 ± 0.78	4.5 ± 0.5	4.43 ± 0.73	4.14 ± 0.64	4.36 ± 0.69	88	100
	N17	4.29 ± 0.72	4.06 ± 0.8	4.29 ± 0.7	4 ± 1.07	4.14 ± 0.35	4.11 ± 0.92	82	98
Net benefits	N18	4.24 ± 0.63	4.12 ± 0.87	4.43 ± 0.49	4.57 ± 0.73	4.43 ± 0.49	4.26 ± 0.72	88	98
	N19	4.21 ± 0.66	4.15 ± 0.91	4.43 ± 0.62	4.43 ± 0.73	4.29 ± 0.45	4.24 ± 0.75	87	97
	N20	4.11 ± 0.72	4.15 ± 0.81	4.36 ± 0.48	4 ± 0.76	4.43 ± 0.49	4.17 ± 0.73	85	98

Abbreviation: SD, standard deviation.

Furthermore, as for user satisfaction, the analysis demonstrates consistently high user satisfaction (81–92%) and strong agreement (97–100%) across all evaluated CPCS-NHIS dimensions, with peak satisfaction observed for nutritional flagging accuracy (N6: 91%) and clinical decision support utility (N15: 92%). Notably, nutrition plan maintenance (N4) and audit trail functionality (N12) achieved perfect satisfaction (100%), reflecting their critical role in user workflows. While clinicians reported marginally lower agreement on information completeness (N7: 98%) and efficiency gains (N19: 97%), the overall results indicate robust system acceptance.

### Reliability and Validity Analysis of the Questionnaire

Furthermore, we performed the reliability and validity analyses of the designed questionnaires. Specifically, Cronbach's α coefficients were 0.85, 0.80, 0.86, 0.81, 0.88, and 0.88 for the respective dimensions, with an overall α of 0.96, indicating strong internal consistency. The KMO measure was 0.93 (>0.60), and the *p* -value obtained through Bartlett's test was <0.05, indicating that the questionnaire data were suitable for factor analysis. Confirmatory factor analysis revealed that all the average variance extracted values were >0.50 and that all the construct reliability values were >0.80, indicating excellent extraction of measures within the factors and high convergent validity. In addition, the chi-square to degrees of freedom ratio was 1.86 (<3), the root mean square error of approximation was 0.09 (<0.10), the root mean square residual was 0.02 (<0.05), and the comparative fit index was 0.92 (>0.90), demonstrating that the model set by the questionnaire exhibited a good fit.

## Discussion

The findings of this study underscore the critical role of a comprehensive CPCS-NHIS in addressing malnutrition among hospitalized patients. Specifically, we tailored insights from prior research to align with the specific operational needs of the construction of the hospital NHIS. 20 21 22 23 Through extensive discussions with multiple related multiple departments, we refined the business processes while considering the workflow of each staff role, system usability, and actual clinical demands, collaboratively developing the HIS, EHR system, and MNS. This collaborative effort led to the development of an integrated CPCS-NHIS designed to support the inpatients requiring nutritional care. Unlike previous nutrition-related information systems, 21 22 the CPCS-NHIS offers notable strengths, including seamless integration with hospital workflows through 14 interface expansions ( Table 1 ), making CPCS-NHIS reproducible in most hospitals, and enabling electronic nutrition medical records, addressing the gaps in traditional EHRs, and enabling clinicians to document nutritional data directly at the point of care without reliance on stationary workstations, thereby enhancing operational flexibility. This integrated design positions the CPCS-NHIS as a potential solution for bridging nutritional care gaps, with future studies needed to evaluate its actual effect on data management efficiency and care team communication.

Moreover, the application of the revised DeLone and McLean IS success model for evaluating the CPCS-NHIS provides a robust framework for assessing system success. When applying the revised DeLone and McLean IS Success model to assess the CPCS-NHIS, we observed consistently high scores across the six evaluation dimensions, reflecting overall user satisfaction with the system and a willingness to continue using it. These high reliability and validity scores indicate that the CPCS-NHIS not only meets the functional needs of health care providers but also enhances user satisfaction. The positive feedback regarding the nutritional plan developed by dietitians reinforces the system's value in clinical practice. Notably, medical staff prioritized the system's capacity to enhance clinical decision-making and work efficiency—over routine task completion—revealing their fundamental focus on professional skill advancement and superior patient outcomes.

Furthermore, the findings of the present study provide a homogeneous management 39 of nutritional care by embedding universal screening and assessment tools such as NRS2002 and PG-SGA, eliminating the subjective differences in manual tool selection, and as a baseline for related system construction. Finally, it lays the groundwork for integrating information technology to overcome challenges associated with transitioning to value-based care. 40

However, challenges such as limited access control for enteral nutrition orders, 41 and usability of the system interface 42 were identified. Addressing these issues is vital for further optimizing the CPCS-NHIS, as they indirectly affect the quality and continuity of care. 43 Furthermore, exploring the potential of CPCS-NHIS to improve the efficiency of clinical nutrition management in different hospital settings, leveraging pertinent research 44 to enhance mobile platform functionalities for closed-loop nutritional service referrals between patients and social and medical care providers, and facilitating continuity of nutritional therapy beyond hospital settings, are valuable research direction in the future. Finally, future iterations of the system should focus on enhancing access control mechanisms and promoting interprofessional collaboration to ensure that all health care team members can contribute effectively to patient care.

## Conclusion

The construction of CPCS-NHIS offers a practical framework for enhancing NCP implementation, providing a benchmark for hospitals seeking to streamline nutrition management through integrated, cross-professional solutions. By facilitating collaboration among dietitians, nurses, clinicians, dispensers, and administrators, and aligning with the NCP, the CPCS-NHIS integrates with existing clinical systems to streamline workflows while maintaining familiar operational patterns. This design reduces barriers to adoption and positions the system as a potential model for other hospitals developing nutrition-focused information systems. Evaluation using the revised DeLone and McLean IS Success Model demonstrated the success of the CPCS-NHIS. The strong user acceptance and system functionality, affirmed its capacity to support clinical nutrition management. The study's findings highlight the CPCS-NHIS as a practical tool for enhancing NCP implementation, offering insights for advancing hospital nutrition care.

## Clinical Relevance Statement

The NHIS created through the cross-professional and cross-sectoral model (CPCS) helps health care providers manage nutritional care for hospitalized patient. By integrating various medical systems, the CPCS-NHIS improves communication among health care professionals, dietitians, and patients, facilitating more personalized and timely nutritional interventions. Additionally, the evaluation using the revised DeLone and McLean IS Success model shows that the CPCS-NHIS is successful. The clinical users are satisfied with the CPCS-NHIS and are likely to continue using and recommending it, indicating its positive impact on the quality and efficiency of health care services.

## Multiple-Choice Questions

1. What is the significance of constructing the CPCS-NHIS?
  - To reduce hospital costs by minimizing the use of nutritional support therapy.
  - To facilitate early and appropriate nutritional management, ultimately improving patient outcomes.
  - To replace all existing medical systems with a single nutritional health care system.
  - To increase the prevalence of malnutrition among hospitalized adults.
  **Correct Answer** : The correct answer is option b. The CPCS-NHIS can realize multiple objectives, such as electronic prescription management, digital nutrition documentation, and data interoperability, all of which enhance the operational efficiency of medical staff. However, the primary significance is to enable health care professionals to promptly assess patients' nutritional status through the utilization of information technology, ensuring that they receive timely early and appropriate nutritional management, ultimately improving patient outcomes.
2. Which model can comprehensively evaluate the success of NHIS based on CPCS?
  - The Moore's Model for Information System Success.
  - The Technology Acceptance Model.
  - The Unified Theory of Acceptance and Use of Technology.
  - The DeLone and McLean IS Success model.
  **Correct Answer** : The correct answer is option d. To evaluate the success of the constructed CPCS-NHIS, we utilized a revised DeLone and McLean IS Success model to assess the system from six dimensions: system quality, information quality, service quality, user satisfaction, intention to use, and net benefits.
3. What does CPCS stand for in the context of the CPCS-NHIS?
  - Cross-professional and cross-sectoral.
  - Clinical practice and care standards.
  - Computerized patient care system.
  - Comprehensive patient care services.
  **Correct Answer** : The correct answer is option a. The CPCS stands for cross-professional and cross-sectoral. The nutrition care process includes nutrition assessment, diagnosis, intervention, monitoring, and evaluation, all of which require a diverse range of professional knowledge. Furthermore, the application of NHIS also covers multiple department and involves collaboration among various health care professionals. Therefore, for the development of NHIS, it is necessary to integrate the requirements and knowledge of relevant departments and stakeholders.
4. Which of the following is a function of the CPCS-NHIS?
  - Automated inventory counting for nutritional supplies.
  - Bedside nutritional risk screening and nutritional assessment.
  - Paper-based prescription issuance for nutritional interventions.
  - Manual collection of patient-reported data for nutritional care.
  **Correct Answer** : The correct answer is option b. The functions of CPCS-NHIS include bedside nutritional risk screening, nutritional assessment, nutritional intervention, diagnosis, enteral nutrition prescription, and QR code payment.
