# Supplementary material for: Development and Evaluation of an Integrated Nutritional Health Care Information System
Source: Appl Clin Inform. 2025 Nov 20;16(5):1749–60. doi: 10.1055/a-2723-6679 (PMC12634206; doi:10.1055/a-2723-6679)

# Functional Screenshots and Descriptions of the CPCS-NHIS

1、Nurses perform nutritional risk screenings for the patient by logging into the MNS on a personal digital assistant

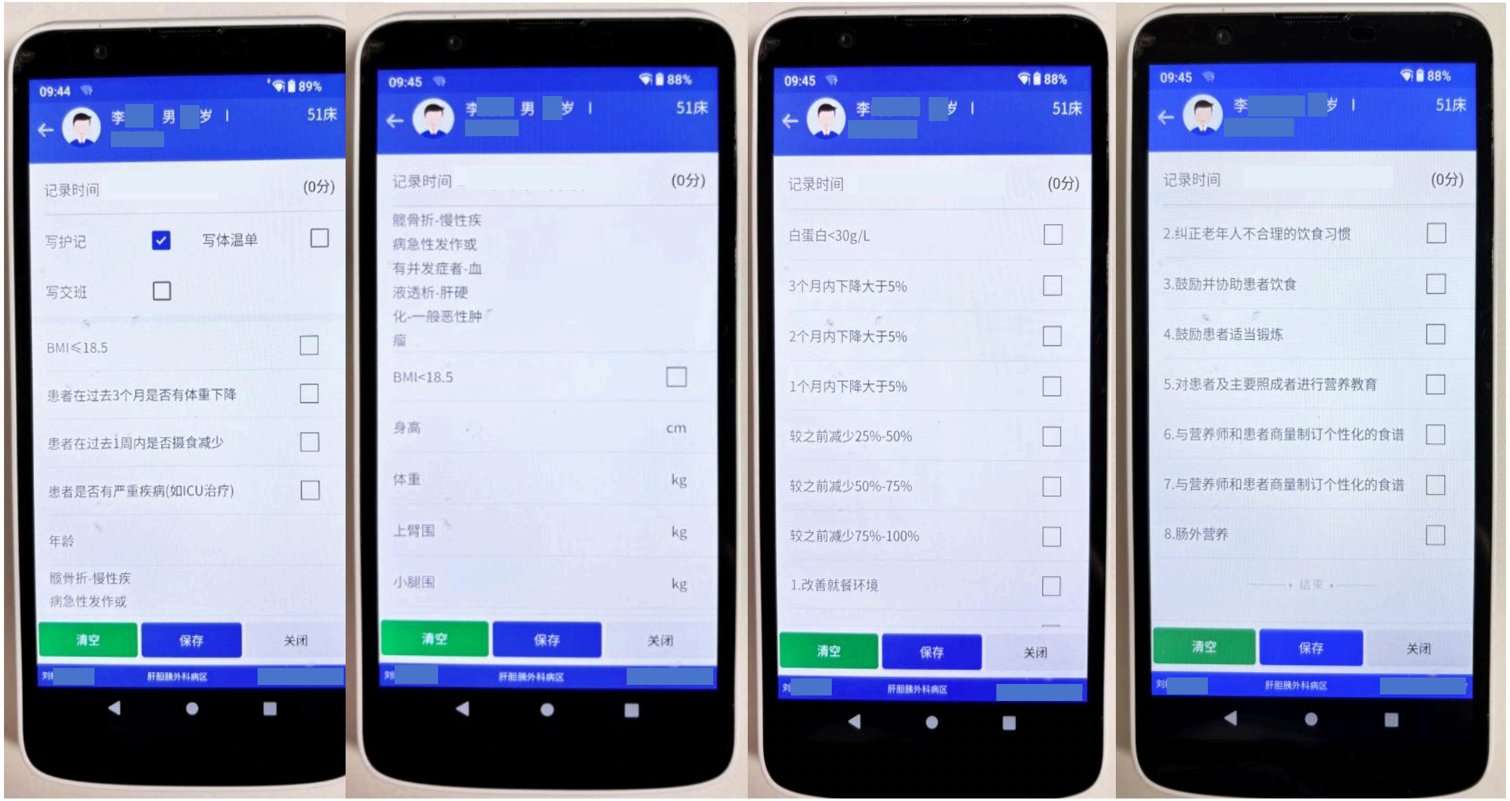

2、When the nutritional risk screening score is≥3, the patient's name in the HIS patient list will be marked with the identifier"Screen".

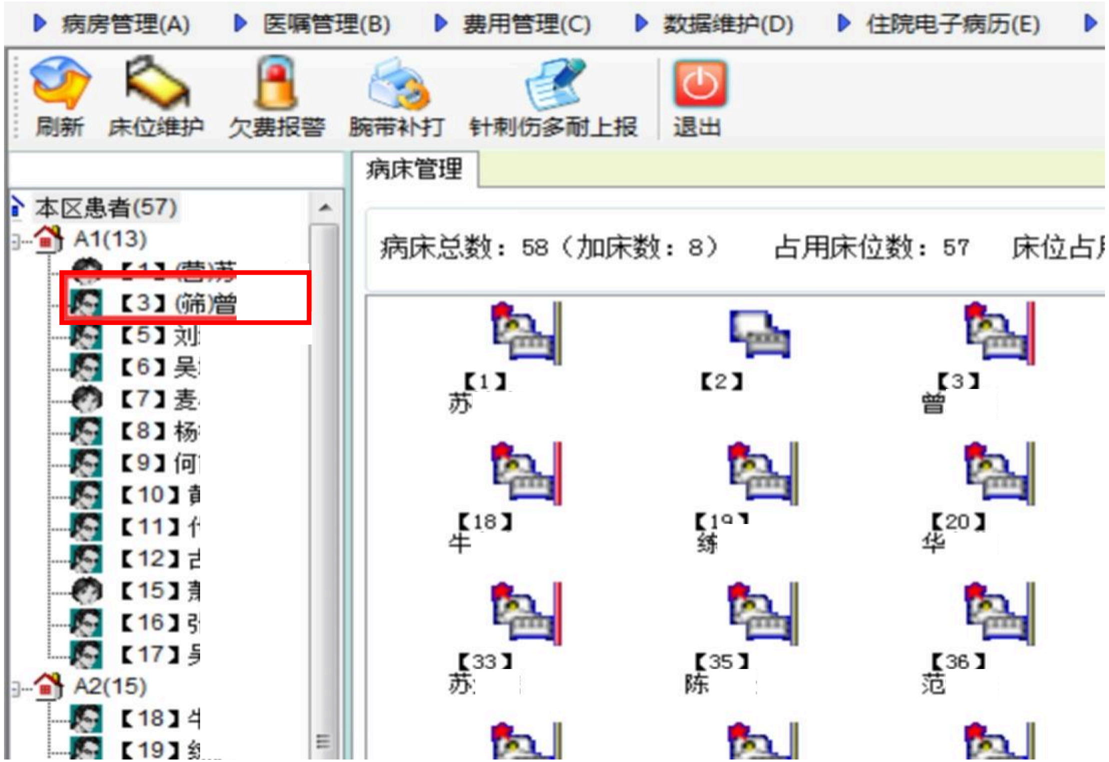

3、Forms for nutritional intervention plans created by clinical dietitians.

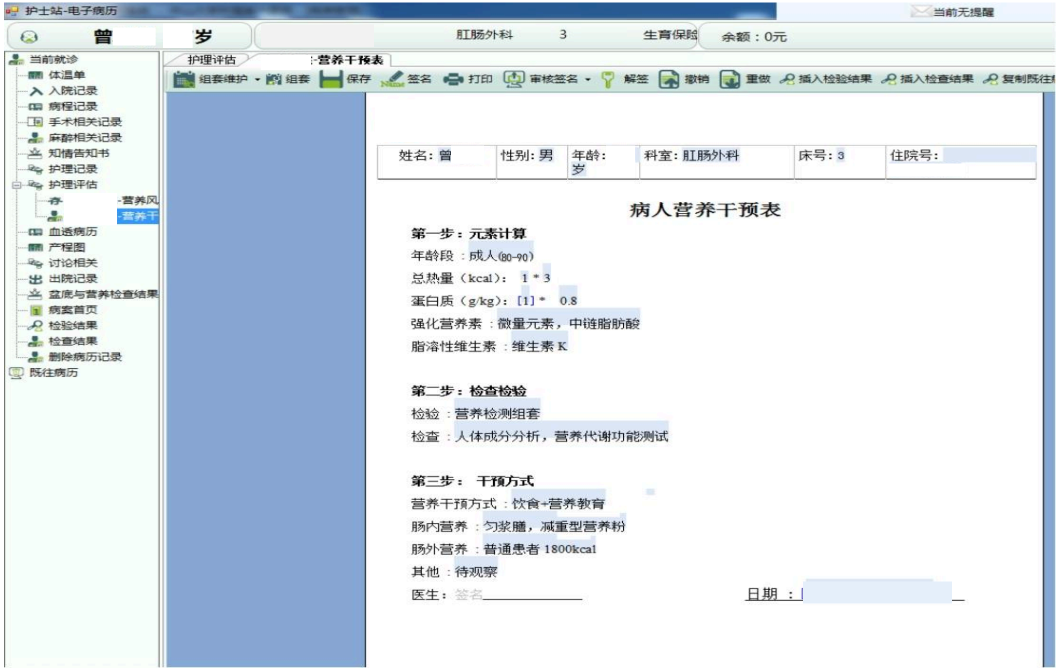

4、When the nutritional intervention plans is created developed, the patient's status in the HIS is updated from 'screened' to 'malnourished'

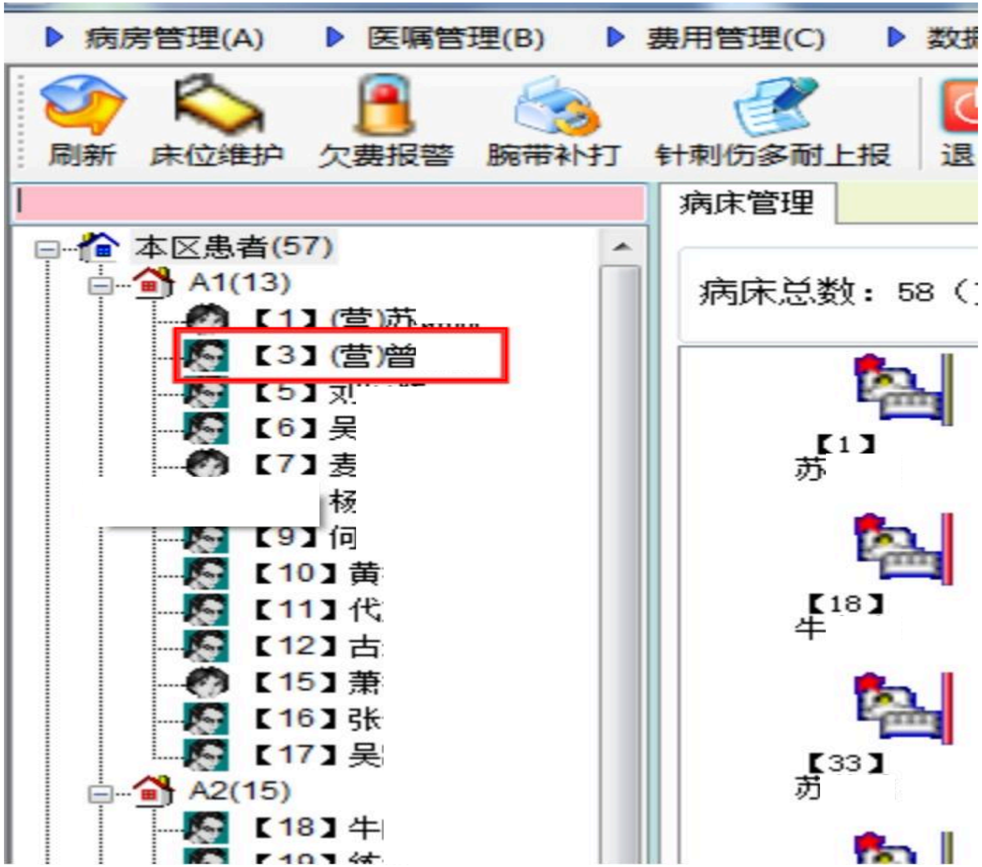

5、The button for issuing nutritional orders for malnourished patients and the button for viewing nutritional intervention plans.

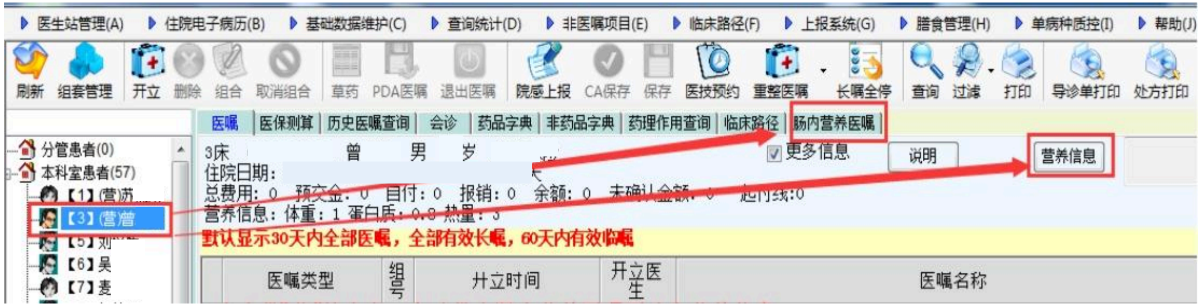


6、When a clinician authorized to allow nutritional prescribing issues a nutritional order for a patient with malnutrition identified as ‘malnourished’ for the first time, the HIS automatically displays the relevant nutritional intervention plan for the clinician’s reference.

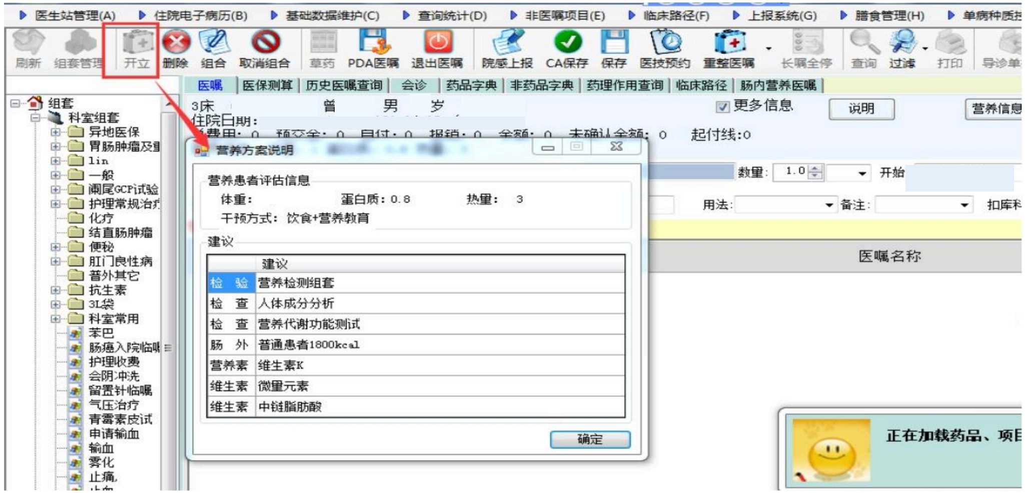

Thieme

7、Nutrition prescription information and pop-up display of the corresponding prescription instructions.

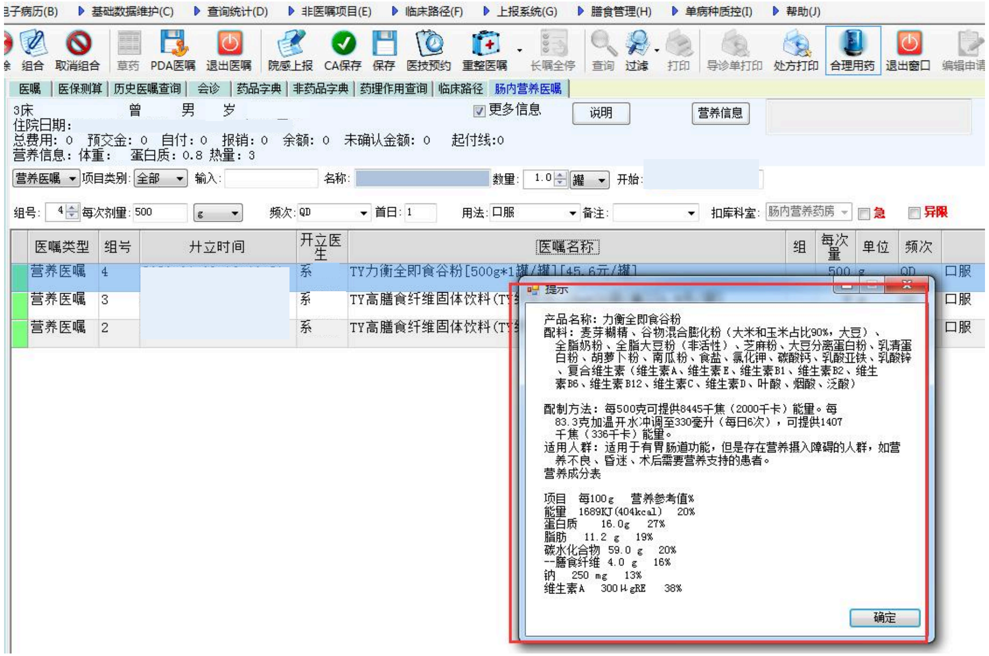

8、A nutritional order form containing patient basic information, nutritional orders, and other relevant information.

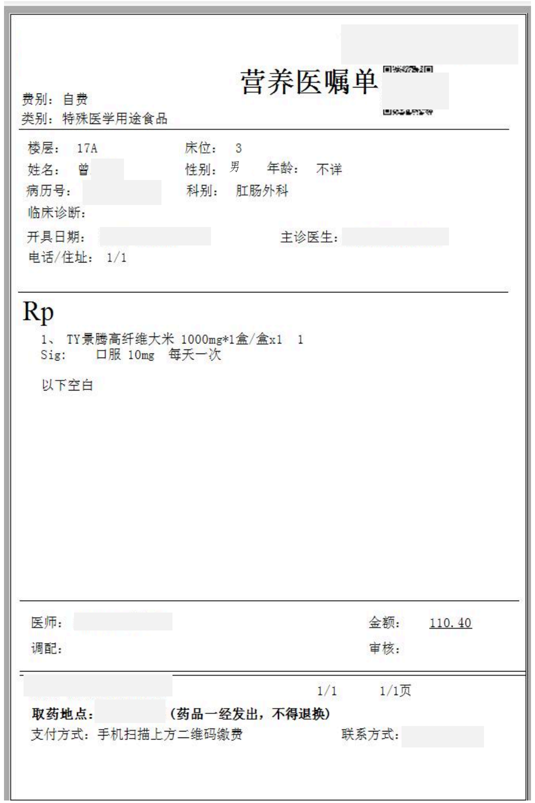

营养医嘱单

费别：自费
类别：特殊医学用途食品

楼层：17A　床位：3
姓名：曾　性别：男　年龄：不详
病历号：　科别：肛肠外科
临床诊断：
开具日期：　主诊医生：
电话/住址：1/1

Rp

1、TY景腾高纤维大米 1000mg*1盒/盒x1　1
Sig:　口服 10mg　每天一次

以下空白

医师：　金额：110.40
调配：　审核：

1/1　1/1页

**取药地点：（药品一经发出，不得退换）**
支付方式：手机扫描上方二维码缴费　联系方式：

9、Patients can scan to pay and view order details, including the amount, payment method, pick-up location, and other information.

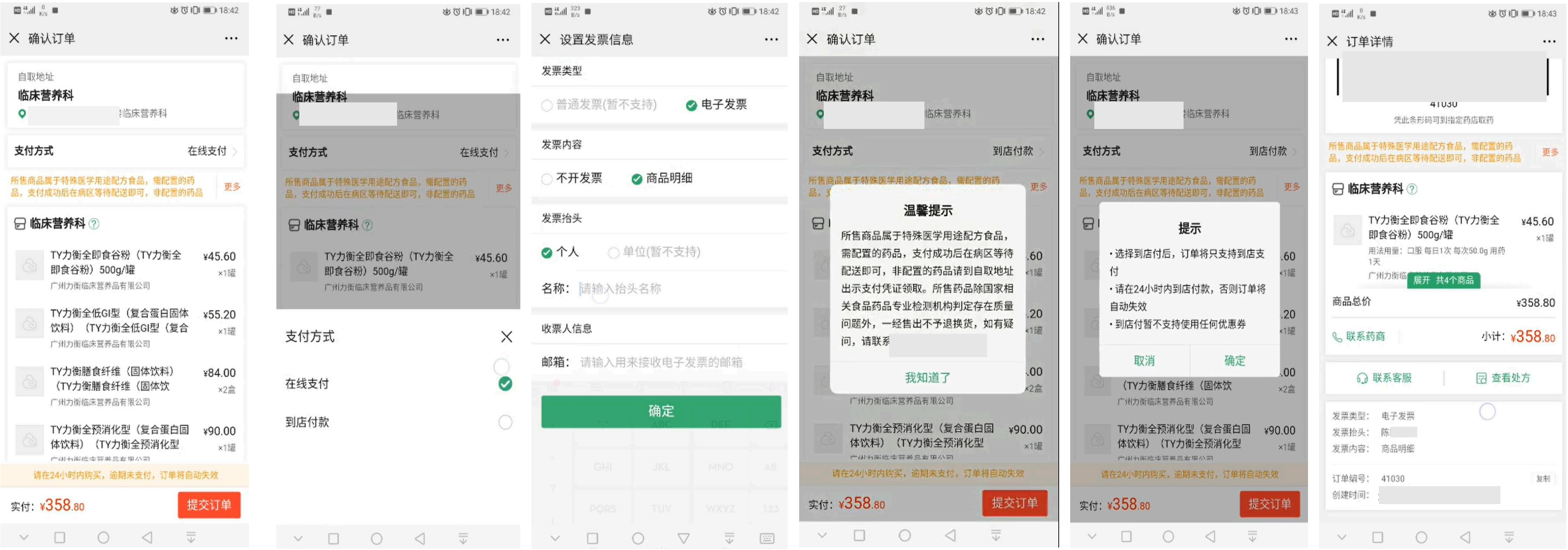

Supplement: Supplementary file 1 — Supplementary Material [file 10-1055-a-2723-6679_27512226.pdf]
